# Effects of Breeder Age and In Ovo Administration of Vitamin D_3_ Metabolites on Hatchability, Growth Performance, Bone Quality, and Leg Health in Broilers

**DOI:** 10.3390/ani16142229

**Published:** 2026-07-18

**Authors:** Zeynep Yardım, İhsan Bülent Helva, Mustafa Akşit

**Affiliations:** 1 Department of Animal Science, Faculty of Agriculture, Aydın Adnan Menderes University, 09970 Aydın, Türkiye; zkacamakli@adu.edu.tr; 2 Çine Vocational School, Aydın Adnan Menderes University, 09520 Aydın, Türkiye; bhelva@adu.edu.tr

**Keywords:** broiler, breeder age, in ovo, vitamin D_3_, 25(OH)D_3_, 1,25(OH)_2_D_3_, tibia, femur, bone mineralization, tibial dyschondroplasia

## Abstract

This study evaluated the effects of breeder age (BA) and in ovo (IO) administration of vitamin D_3_ metabolites on hatchability, body weight, bone traits, and leg health in broiler chickens. A total of 640 eggs obtained from young (YB; 29 wk) and old (OB; 52 wk) Ross 308 broiler breeder flocks were assigned on day 18 of incubation to four treatments: 25(OH)D_3_, 1,25(OH)_2_D_3_, diluent, or non-injected. Chicks were reared under standard conditions until market age. Hatchability was not affected by BA or IO treatments. Birds derived from OBs exhibited greater hatch weight and final body weight than those derived from YBs. At market age, BA effects on bone traits were limited to greater femur length in OB birds. In contrast, IO administration of vitamin D_3_ metabolites significantly enhanced bone mineralization and mechanical competence, as evidenced by higher tibial Ca, P, and Mg concentrations; increased bone ash content; a higher Seedor index; and greater breaking strength. Treated birds also exhibited shorter, narrower, and heavier long bones, indicating altered bone geometry. Furthermore, the incidence of gait abnormalities and tibial dyschondroplasia was markedly reduced, whereas femoral head necrosis remained unaffected. Among the metabolites evaluated, 25(OH)D_3_ produced the most consistent improvements in final body weight, bone traits, and leg health. Overall, IO administration of vitamin D_3_ metabolites, particularly 25(OH)D_3_, improved bone traits and leg health at market age and may represent a practical strategy to enhance broiler welfare and production performance.

## Simple Summary

Skeletal deformities and leg problems are major welfare and economic concerns in broiler production. This study evaluated whether breeder age and in ovo administration of vitamin D_3_ metabolites affect growth, bone development, and leg health in broiler chickens. Vitamin D_3_ metabolites were injected into eggs during incubation, and birds were reared to market age. Breeder age had limited effects on bone traits, whereas in ovo vitamin D_3_, particularly 25(OH)D_3_, improved bone quality and leg health. These effects were associated with enhanced bone strength and reduced gait abnormalities and tibial dyschondroplasia, suggesting a practical strategy to improve broiler skeletal welfare.

## 1. Introduction

Genetic selection for rapid growth and high meat yield in broiler chickens has significantly improved production efficiency; however, it has also led to major problems such as skeletal disorders, gait abnormalities, and reduced locomotor capacity [1,2]. These conditions are among the leading causes of carcass condemnation and mortality in modern broiler production systems [3]. This imbalance between muscle and skeletal development particularly compromises the structural integrity of long bones during early growth [4]. Skeletal development begins during the embryonic period, which accounts for approximately one-third of the broiler life cycle. During this stage, bone formation depends largely on nutrient reserves within the egg [5]. Calcium and phosphorus are mobilized from the yolk during early incubation and from the eggshell during late incubation, contributing to bone mineralization [6,7]. Vitamin D_3_ metabolism plays a central role in embryonic calcium and phosphorus homeostasis. Vitamin D_3_ is first hydroxylated in the liver to 25-hydroxyvitamin D_3_ (25(OH)D_3_) and subsequently converted in the kidney into the biologically active form 1,25-dihydroxyvitamin D_3_ (1,25(OH)_2_D_3_) [8]. Together, these metabolites regulate mineral absorption and osteogenic activity [9,10,11]. Therefore, hormonal regulation of embryonic mineral utilization represents a critical control point for skeletal development.

Breeder age (BA) is an important factor affecting egg composition and embryonic development. Eggs from older breeders (OBs) are generally heavier and contain higher mineral concentrations (Ca, P, and Mg) [12,13]. In contrast, eggs from young breeders (YBs) may have lower nutrient reserves, potentially limiting embryonic development [7,14]. Magnesium is rapidly utilized during early embryogenesis and plays an important role in bone matrix formation [15]. In addition, its role as a cofactor in vitamin D metabolism suggests a potential interaction with BA effects on mineral utilization efficiency.

In ovo administration (IO) of vitamin D_3_ during incubation has been proposed as a strategy to improve embryonic mineral status and skeletal development [16,17]. In particular, 25(OH)D_3_ is a more stable form and enhances calcium utilization [18,19]. The late embryonic period represents a critical window in which calcium mobilization from the eggshell reaches its peak [15]. However, whether breeder age-related differences in embryonic mineral utilization translate into persistent differences in post-hatch skeletal development, and whether these responses can be modified by IO D_3_ supplementation, remains unclear.

Previous studies have demonstrated beneficial effects of in ovo vitamin D supplementation on embryonic development, post-hatch performance, and selected indicators of skeletal development. However, limited information is available regarding whether the skeletal responses to in ovo vitamin D_3_ metabolite supplementation are influenced by breeder age, despite well-established differences in egg composition and embryonic nutrient availability between young and old breeder flocks. It was hypothesized that IO administration of D_3_ metabolites would enhance bone mineralization and promote tibial and femoral development and that these responses might vary according to breeder age-related differences in embryonic nutrient and mineral reserves. Therefore, this study investigated the effects of IO administration of D_3_ metabolites on tibial and femoral morphometric traits, breaking strength, and mineral composition (Ca, P, and Mg) in broilers derived from YB and OB flocks. Hatchability, growth performance, and leg health indicators, including gait score (GS), tibial dyschondroplasia (TD), and femoral head necrosis (FHN), were also evaluated.

## 2. Materials and Methods

### 2.1. Ethical Statement

The experimental protocol was approved by the Ethical Committee for the Care and Use of Experimental (2020/90 and 2022/128) of Aydın Adnan Menderes University.

### 2.2. Hatching and Treatments

A total of 640 broiler eggs (Ross 308) obtained from YB (29 weeks) and OB (52 weeks) flocks from a commercial breeding company (Lezita Inc., İzmir, Türkiye) were used in the study. On the day of lay, 320 hatching eggs from the YB flock (59.6 ± 2 g) and 320 from the OB flock (67.3 ± 2 g) were transported to the hatchery unit in a sterile transport cabin maintained at 20–22 °C and 50–55% relative humidity (RH).

The incubation and growing periods were conducted at the Aydın Adnan Menderes University (ADU) Poultry Unit of the Faculty of Agriculture, and meat and bone analyses were performed at ADU TARBIYOMER laboratories (Aydın, Türkiye). Prior to setting, eggs were stored for 1 day at 20 ± 2 °C and 50–55% RH. The eggs were individually weighed and randomly assigned in equal numbers (in each group, 80 YB eggs and 80 OB eggs) to four experimental groups:

(a) 25-hydroxyvitamin D_3_ (25(OH)D_3_: Calsifediol (Cat. No.: HY-32351, MedChemExpress, Monmouth Junction, NJ, USA)) dissolved in saline was injected into the amniotic fluid on E18 at a dose of 0.60 µg per egg in a volume of 100 μL containing 0.12% dimethyl sulfoxide (DMSO; Sigma-Aldrich/Merck KGaA, Darmstadt, Germany).
(b) 1,25-dihydroxyvitamin D_3_ (1,25(OH)_2_D**_3_**: Calcitriol, (Cat. No.: HY-100022, MedChemExpress, Monmouth Junction, NJ, USA)) dissolved in saline was injected into the amniotic fluid on E18 at a dose of 0.60 µg per egg in a volume of 100 μL containing 0.12% DMSO.
(c) Diluent: A total of 100 µL of saline containing 0.12% DMSO was injected into the amniotic fluid on E18 as the diluent.
(d) Non-injected: Eggs in this group were not injected

Eggs were set in an incubator (Nestbox, NB 348, Nestbox, Kütahya, Türkiye) at a density of 20 eggs per tray under standard conditions: 37.8 ± 0.1 °C and 55% RH with automatic turning every 60 min from day 0 to 18, followed by 36.8 ± 0.1 °C, 70% RH, without turning from day 19 until hatch. Incubator air temperature and RH were continuously monitored with Wi-Fi sensors (TFA-WeatherHub; Dostmann GmbH & Co. KG, Wertheim-Reicholzheim, Germany) during the incubation.

At 18 d of incubation (432 h), fertility was assessed by candling, and infertile eggs were recorded. IO administration of vitamin D_3_ metabolites into the amniotic fluid of fertile eggs was performed using sterile disposable 1 mL syringes (100 µL per egg), following [17]. The dose of 0.60 µg/egg was selected based on previous in ovo vitamin D studies demonstrating its safe application and physiological efficacy in broiler embryos [16,17]. Although 25(OH)D_3_ and 1,25(OH)_2_D_3_ differ markedly in biological potency, the same nominal dose of both metabolites was administered to enable a standardized comparison of their physiological effects under identical administration conditions (i.e., equal injection volume and vehicle concentration), rather than to establish biologically equivalent dosing. Before injection, shells were disinfected with 70% ethanol; a small hole was drilled with a sterile pin without damaging the outer shell membrane, the solution was delivered into the amniotic fluid, and the hole was sealed with melted paraffin wax. Following IO, the eggs were transferred to the hatcher (Nestbox NB-348, Kütahya, Türkiye).

A total of 494 (242 YB–252 OB) chicks hatched in all groups between 492 and 512 h of incubation. Fertility (%) was calculated as the number of fertile eggs/total number of eggs set × 100. The hatchability of fertile eggs (%) was calculated as the number of hatched chicks/number of fertile eggs × 100.

### 2.3. Growth Performance

The growing period was conducted with 448 randomly selected chicks. After hatching, the chicks were wing-banded and weighed, then they were allocated to 32 floor pens (2 BA × 4 IO treatments × 4 replicates—14 birds per pen) randomly. The birds were reared under standard management (14 birds/m^2^) until 42 days of age. Diets were chosen according to Ross 308 nutrition recommendations [20]. Lighting was 23L:1D during days 0–7 and 16L:8D thereafter. The temperature was 33 °C during the first three days and then was decreased by 3 °C every week until an ambient temperature of 24 °C was reached. Feed and water were provided ad libitum. Mortality was recorded daily. On days 21 and 42, all birds were weighed, and pen-level feed intake was recorded; the feed conversion ratio (FCR) was calculated as the feed intake divided by body weight (BW) for the corresponding period.

### 2.4. Bone Assessments

On day 42, 12 broilers per group (6 males and 6 females, totaling 96 broilers) were weighed and slaughtered by being rendered unconscious with electrical stunning according to EFSA [21] recommendations (120 mA per bird, 5 s, AC 50 Hz, sinusoidal type) to assess tibia and femur characteristics using standard procedures. The soft tissues were removed from the tibias and femurs of the broilers, and the bones were dried at 60 °C for 72 h in a drying oven. Then they were weighed. The diameter and length of the tibia and femur bones were measured using a digital caliper (Mitutoyo 500-181-30, Mitutoyo Corporation, Kawasaki, Japan), and the breaking strength (Newton, N) was determined in a Zwick/Roell Z 50 (ZwickRoell GmbH & Co. KG, Ulm, Germany) testing machine (TextXpert Version 3.4) with the Warner–Bratzler method. The Seedor index (SI) and robusticity index (RI) of tibia and femur bones were calculated (SI = bone weight (mg)/bone length (mm), RI = bone length/cube root of bone weight (g)) [22,23,24]. The ash contents of the left tibia and femur of each bird were determined on a fat-free dry weight basis, according to AOAC International (2005; method 932.16) [25] procedures. After cooling, only the tibial ash samples were subjected to mineral analysis. For this purpose, 10 mL of 1% HNO_3_ was added to each tibial ash sample for mineral extraction. The extracts were filtered through Whatman No. 1 filter paper and diluted to 50 mL with deionized water. Calcium (Ca), phosphorus (P), and magnesium (Mg) concentrations were determined only in the tibia by ICP-OES [26] using certified multi-element calibration standards.

### 2.5. Leg Health Assessments

On day 39, GS was evaluated in all broilers according to Webster et al. (2008) [27] (0: no impairment of walking ability; 1: having obvious impairment but still ambulatory; 2: having severe impairment and not able to walk without great difficulty). On day 42, at slaughter, the left and right tibias and femurs were collected and macroscopically evaluated for tibial dyschondroplasia (TD) and femoral head necrosis (FHN), respectively. For TD assessment, the tibias were longitudinally sectioned and scored according to the method described by Edwards and Veltmann (1983) [28], where scores were defined as 0 = normal (no apparent TD), 1 = growth plates 1 to 2 mm wide, 2 = growth plates 2 to 3 mm wide, and 3 = growth plates greater than 3 mm wide. The femurs were subsequently evaluated for the severity of FHN lesions and scored on a scale ranging from 0 to 3 according to Wiemer et al. (2021) [29], where 0 = normal (no abnormalities), 1 = separation of the femoral head from the articular cartilage, 2 = transitional degeneration, and 3 = severe necrosis. All GS, TD, and FHN assessments were performed by the same experienced evaluator, who was blinded to treatment allocation throughout the evaluations.

### 2.6. Statsitical Analysis

Sample size was determined using G*Power (version 3.1.9.7) for a 2 × 4 factorial design [30]. Standard criteria were applied: α = 0.05, statistical power (1 − β) = 0.95, and a medium effect size (f = 0.25). Although the analysis indicated a minimum requirement of 70 eggs per group, 80 eggs were used to enhance experimental robustness. The experimental and statistical units varied according to the response variable. For hatchability and incubation traits, the incubation tray (replicate) was considered the experimental unit. Body weight was analyzed using the individual bird as the statistical unit. Feed intake and feed conversion ratio were analyzed using the pen (replicate) as the experimental unit. Bone characteristics, tibial mineral composition, gait score, tibial dyschondroplasia, and femoral head necrosis were analyzed using the individual bird as the statistical unit. Equal numbers of male and female birds (6 males and 6 females per treatment group) were sampled for all individual measurements. Sex was initially included in the statistical model as an additional fixed effect. However, because neither the main effect of sex nor its interactions with the experimental factors were statistically significant (*p* > 0.05), sex was excluded from the final model. Eggs were randomly assigned to treatments prior to incubation, and hatchability rates were considered in the final statistical analyses. Initially, sex was incorporated into the statistical model as a main factor to assess its potential influence and interactions on 42-day bone parameters. However, because neither the main effect of sex nor its interactions with the experimental factors were statistically significant (*p* > 0.05), male and female data were pooled for the final analyses. Data for all variables were analyzed using the IBM SPSS 18.0 software package program (SPSS Inc., Chicago, IL, USA) [31]. Normality analysis was conducted to assess the distribution characteristics of the residuals from the mixed model analyses.

Growth performance, tibia and femur characteristics, tibial mineral composition, and breast meat quality traits were analyzed using the GLM (General Linear Model) procedure according to the following two-way factorial model:

*Y_ijk_ = μ + BA_i_ + IO_j_ + *(*BA × IO*)*_ij_ + e_ijk_* (1)

where *Y_ijk_* is the observed value, *μ* is the overall mean, *BA_i_* is the fixed effect of breeder age, *IO_j_* is the fixed effect of in ovo D_3_ metabolite treatment, (*BA × IO*)*_ij_* is the interaction between breeder age and in ovo D_3_ metabolite treatment, and *e_ijk_* is the random error term. Statistical differences among treatment means were determined using Tukey’s multiple comparison test at *p* < 0.05. Gait score, tibial dyschondroplasia and femoral head necrosis in broilers were analyzed using a non-parametric chi-square (χ^2^) test.

## 3. Results

### 3.1. Hatching Performance

The effects of BA and IO administration of D_3_ metabolites on egg weight, fertility and hatching performance are presented in Table 1. BA significantly influenced egg weight, with eggs from the OB group being heavier than those from the YB group (*p* < 0.05), whereas IO treatments had no effect on egg weight (*p* > 0.05). Fertility, hatchability of fertile eggs and embryonic mortalities (late and total) were not affected by BA and IO treatments (*p* > 0.05). No significant interaction was determined between BA and IO for any traits (*p* > 0.05).

### 3.2. Growth Performance

Table 2 presents the BW, feed consumption (FC), and FCR of the birds during the experimental period. BA significantly affected the BW of chicks (*p* < 0.05). Chicks from the OB group had a higher BW at hatch (initial chick weight) as well as at 21 and 42 days compared with those from the YB group (*p* < 0.05; Table 2). Similarly, FC was affected by BA, with OB chicks consuming more feed than YB chicks throughout all periods (*p* < 0.05). IO administration of 25(OH)D_3_ and 1,25(OH)_2_D_3_ increased BW at both 21 and 42 days of age compared to the diluent and non-injected groups (*p* < 0.05). FC was higher in broilers that underwent IO treatment with D_3_ metabolites from d 0 to 42 (*p* < 0.05), with no differences during the first 21 d. FCR was not affected by the treatments (*p* > 0.05). There were no significant interaction effects between BA and IO for any of the evaluated parameters (*p* > 0.05; Table 2).

Total mortality over the 42-day period averaged 2.68% and was not affected by the treatments (*p* > 0.05).

### 3.3. Bone Quality

The effects of BA and IO administration of D_3_ metabolites on tibia and femur traits in broilers are presented in Table 3. BA had no significant effect on measured leg bone traits in broilers (*p* > 0.05), except for femur length, which was greater in the OB group (*p* < 0.05). IO administration of 25(OH)D_3_ and 1,25(OH)_2_D_3_ increased tibia and femur weights and breaking strength compared with the diluent and non-injected groups, whereas tibia and femur length was reduced in the metabolite-treated groups (*p* < 0.05).

**Figure 1:**
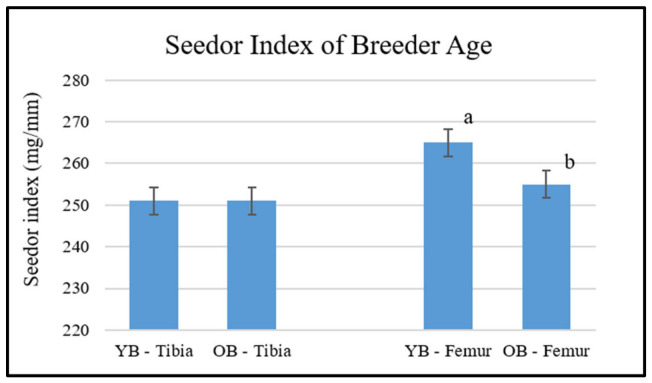
The effect of breeder age on the Seedor index of tibias and femurs. YB, young breeder (29 weeks old); OB, old breeder (52 weeks old). Means within a bar and line with different superscripts (a,b) differ significantly (*p* < 0.05). SEM = 0.001.

**Figure 2:**
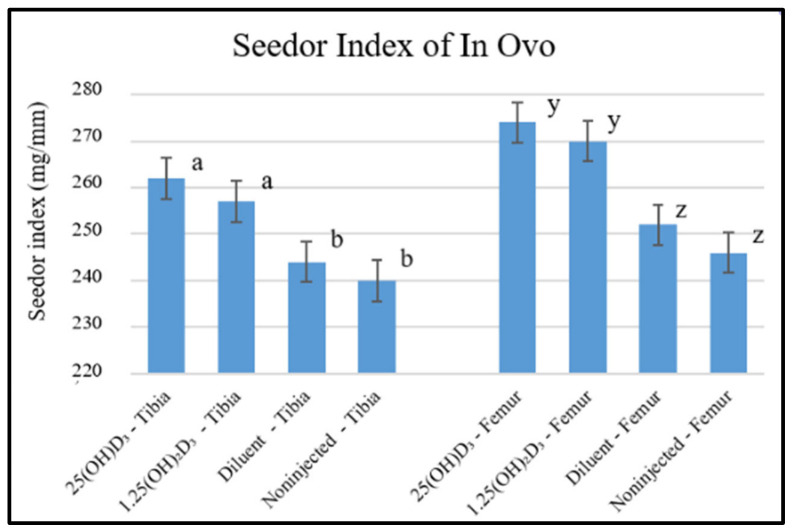
The effect of in ovo administration of vitamin D_3_ metabolites on the Seedor index of tibias and femurs. Means within a bar and line with different superscripts (a,b for tibia and y,z for femur) differ significantly (*p* < 0.05). In ovo: 25(OH)D_3_: Calsifediol dissolved in saline was injected into the amniotic fluid on E18 at a dose of 0.60 µg per egg in a volume of 100 μL containing 0.12% DMSO; 1,25(OH)_2_D_3_: Calcitriol dissolved in saline was injected into the amniotic fluid on E18 at a dose of 0.60 µg per egg in a volume of 100 μL containing 0.12% DMSO. Diluent: A total of 100 µL of saline containing 0.12% was injected into the amniotic fluid on E18 as the diluent. Non-injected: Eggs were not injected. SEM = 0.027.

**Figure 3:**
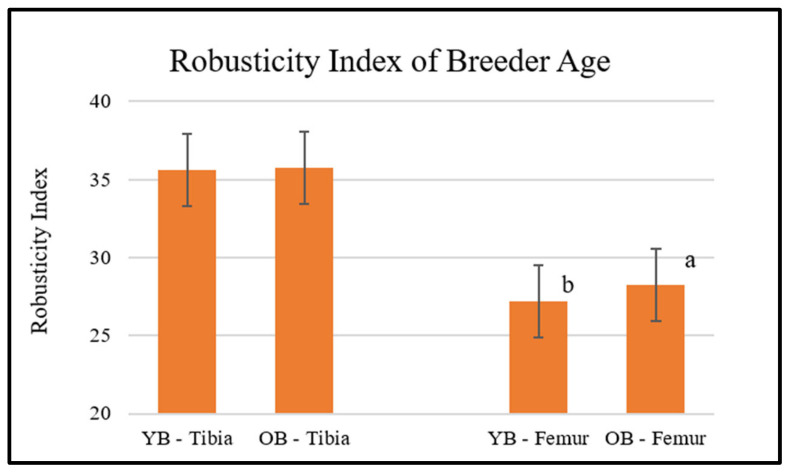
The effect of breeder age on the robusticity index of tibias and femurs. YB, young breeder (29 weeks old); OB, old breeder (52 weeks old). Means within a bar and line with different superscripts (a,b) differ significantly (*p* < 0.05). SEM = 0.001.

**Figure 4:**
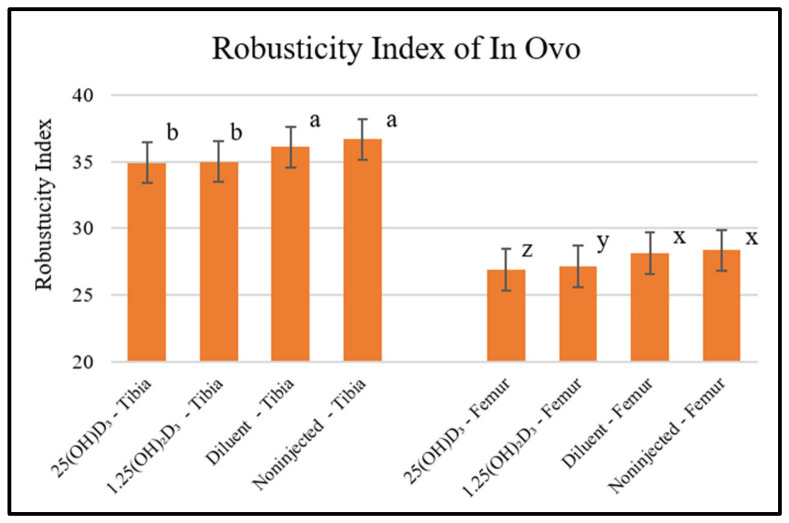
The effect of in ovo administration of vitamin D_3_ metabolites on the robusticity index of tibias and femurs. Means within a bar and line with different superscripts (a,b for tibia and x,y,z for femur) differ significantly (*p* < 0.05). In ovo: 25(OH)D_3_: Calsifediol dissolved in saline was injected into the amniotic fluid on E18 at a dose of 0.60 µg per egg in a volume of 100 μL containing 0.12% DMSO; 1,25(OH)_2_D_3_: Calcitriol dissolved in saline was injected into the amniotic fluid on E18 at a dose of 0.60 µg per egg in a volume of 100 μL containing 0.12% DMSO. Diluent: A total of 100 µL of saline containing 0.12% was injected into the amniotic fluid on E18 as the diluent. Non-injected: Eggs were not injected. SEM = 0.027.

Figure 1, Figure 2, Figure 3 and Figure 4 show the effects of BA and IO administration of D_3_ metabolites on the Seedor index (SI) and robusticity index (RI). BA did not influence the tibia SI and RI (*p* > 0.05, Figure 1 and Figure 3); however, it significantly affected the femur SI and RI (*p* < 0.05). Specifically, OB broilers showed lower femur SI and higher RI compared with their YB counterparts (*p* < 0.05).

IO administration of 25(OH)D_3_ and 1,25(OH)_2_D_3_ increased the SI and decreased RI in both the tibia and femur compared with the diluent and non-injected groups (*p* < 0.05, Figure 2 and Figure 4). No significant interaction between BA and IO was detected for either index.

Table 4 presents the effects of breeder age and IO administration of D_3_ metabolites on the Ca, P, and Mg concentrations of the tibia, as well as the ash content (%) of both the tibia and femur. Tibia Ca, P, and Mg concentrations were higher in YB than in OB broilers (*p* < 0.05). IO administration of 25(OH)D_3_ and 1.25(OH)_2_D_3_ increased tibia Ca, P, and Mg concentrations compared with the diluent and non-injected groups (*p* < 0.05). BA had no significant effect on tibia and femur ash content (*p* > 0.05), whereas IO administration of D_3_ metabolites significantly increased both tibia and femur ash content compared with the diluent and non-injected groups (*p* < 0.05).

### 3.4. Leg Health

Table 5 presents the effects of BA and IO administration of D_3_ metabolites on GS, TD and FHN in broilers. While BA had no significant effect on GS, TD, or FHN (*p* > 0.05), IO administration of D_3_ significantly influenced GS and TD (*p* < 0.05), whereas FHN remained comparable among treatment groups (*p* > 0.05). The distributions of GS, TD, and FHN scores are presented in Figures S1–S3 in the Supplementary Materials. Specifically, the lowest GS and TD incidences were observed in the IO 25(OH)D_3_ group (*p* < 0.05).

## 4. Discussion

### 4.1. Breeder Age

BA is a key determinant of egg characteristics and consequently early embryonic development [32]. In the present study, eggs from OBs were heavier than those from YBs, in agreement with previous reports showing that egg weight increases with advancing BA [32,33,34]. Despite this difference in egg weight, neither BA nor IO administration of D_3_ affected hatchability or embryonic mortality [2,17,35,36,37,38,39]. This suggests that, under the present incubation conditions, embryonic development remained within a physiological range that was not sensitive to moderate differences in BA. Although reductions in hatchability have been associated with advanced BA through changes in shell quality, gas exchange, and antioxidant status [40,41], such effects were not evident here. This may be related to the relatively narrow age difference between the breeder flocks, as BA effects on incubation traits are often inconsistent when age differences are limited [32,42,43]. Taken together, these findings indicate that BA primarily influenced egg size rather than incubation success in the present experiment.

BA had a clear effect on growth performance throughout the rearing period. Chicks originating from OBs were heavier at hatch and maintained this advantage during the growing period, resulting in consistently higher body weights compared with chicks from YBs. This pattern is consistent with previous reports indicating that the greater hatch weight and early developmental advantages of chicks from older breeder flocks tend to persist when early developmental advantages are established prior to hatch [44,45,46]. The most likely explanation for this difference is related to the developmental status of the embryo at oviposition and early nutrient availability. Embryos from OBs generally possess more advanced morphological development and greater yolk nutrient reserves, which may support early post-hatch growth potential [47]. In addition, differences in yolk lipid composition and nutrient density between YBs and OBs may contribute to early growth divergence [48]. These early advantages appear to translate into higher feed intake capacity during the rearing period, which was also consistently higher in OB birds in the present study. Despite higher feed intake in OB birds, the feed conversion ratio was not affected by BA, suggesting that nutrient utilization efficiency remained comparable between groups. This indicates that BA primarily influenced the scale of growth rather than metabolic efficiency.

### 4.2. In Ovo Administration of Vitamin D_3_ Metabolites

IO administration of D_3_ metabolites showed a more treatment-dependent response. While hatch weight was not affected, 25(OH)D_3_ administration resulted in higher body weights at 21 and 42 days compared with the control groups, whereas 1,25(OH)_2_D_3_ did not produce a similar response. This difference may be associated with the more stable circulating profile of 25(OH)D_3_, which provides a sustained substrate pool for downstream conversion into the active hormonal form [49].

Interestingly, the absence of a significant BA × IO interaction suggests that the growth-promoting effects of vitamin D_3_ metabolites were expressed similarly in both BA groups. In other words, vitamin D_3_ supplementation improved growth performance, but it did not preferentially compensate for the lower performance of chicks derived from YBs. This indicates that the mechanisms underlying BA-related growth differences and vitamin D_3_-mediated growth effects may operate largely through independent biological pathways rather than a shared regulatory axis.

Embryos from YBs may have had reduced access to yolk-derived nutrients and mineral reserves during critical stages of skeletal development, as reflected by the lower egg weights observed in this group. Because femoral development involves rapid growth and intensive mineralization during early embryogenesis [50], such nutritional constraints may have limited long bone development. Consistent with this interpretation, broilers derived from YBs exhibited shorter femur lengths than those derived from OBs, in agreement with the findings of Varol Avcılar et al. (2023) [14]. In contrast, BA did not significantly affect tibial length. This likely reflects the delayed mineralization pattern of the tibia, which continues into the late embryonic period [15]. During this stage, calcium mobilized from the eggshell via the chorioallantoic membrane becomes the main mineral source [50], which may partially buffer earlier differences in yolk-derived nutrients. As a result, femur development appears to be more dependent on early nutrient availability, whereas tibial growth is stabilized by late incubation mineral transfer. Despite these structural differences, BA did not affect femur or tibia breaking strength at 42 days of age. This aligns with previous findings showing that early skeletal differences tend to diminish during post-hatch growth [51,52]. Under standard rearing conditions, compensatory growth and remodeling likely mask initial embryonic disparities by processing age.

However, the mineral data reveal a more complex situation. Tibia Ca, P, and Mg concentrations were higher in broilers derived from YBs, yet neither ash content nor mechanical strength were influenced. A similar pattern was also observed in the femur. A similar outcome was observed for femoral ash content and breaking strength, although femoral mineral composition was not determined in the present study. This suggests that increased mineral concentration at the tissue level does not necessarily correspond to greater total mineral deposition or improved mechanical competence. The mechanisms underlying these observations cannot be determined from the present study because bone matrix composition and microstructural characteristics were not evaluated. Several hypotheses may explain these findings. One possible hypothesis is that mineral distribution within the bone matrix may have differed without parallel changes in overall bone mass or geometry. In this context, unchanged SI and RI further suggest that cortical architecture remained stable despite shifts in mineral composition. It is also possible that differences lie in matrix quality rather than mineral quantity. Variations in collagen organization, collagen cross-linking, and organic matrix maturity may influence the relationship between mineral composition and bone mechanical properties without necessarily altering whole-bone ash content or mechanical strength [53,54]. Bone strength reflects the interaction between mineral composition, the organic matrix, and skeletal geometry, indicating that mineral concentration alone is insufficient to explain mechanical competence or overall bone quality [53,54,55]. Alternatively, the apparent discrepancy may reflect the fact that elemental mineral concentrations, whole-bone ash content, and mechanical strength represent different aspects of bone quality and therefore do not necessarily change in parallel [55,56]. Therefore, changes in elemental mineral concentrations may not necessarily be accompanied by proportional changes in whole-bone ash content or mechanical strength, particularly when overall bone architecture remains unchanged. These proposed mechanisms should therefore be regarded as hypotheses rather than direct explanations of the present findings. Accordingly, the present findings support the current understanding that bone quality arises from the interaction of mineralization, matrix characteristics, and structural organization, rather than from changes in any single component alone [54,55]. Because bone characteristics were evaluated only at market age (42 d), the present findings reflect the final status of skeletal mineralization at the end of the growing period and do not provide information on earlier developmental changes; therefore, transient differences in mineral deposition or matrix maturation cannot be excluded. These considerations may help explain why the chemical and mechanical indicators did not fully align in the present study. Although tibia ash percentage did not differ significantly between groups, numerically higher values observed in broilers derived from YBs were consistent with the increased Ca, P, and Mg concentrations. This suggests that elemental mineral deposition may be more sensitive to BA-related variation than total ash content, which represents a more global measure of the inorganic matrix. Because bone ash reflects the total inorganic fraction of bone, whereas elemental analyses quantify individual mineral constituents, these complementary measurements do not necessarily change in parallel, particularly when skeletal traits are evaluated at a single developmental endpoint [56].

Vitamin D_3_ metabolite supplementation, in contrast, consistently improved bone mineralization and mechanical properties across BA groups. The beneficial effects were observed irrespective of breeder age. Based on previous studies, vitamin D metabolites (25(OH)D_3_ and 1,25(OH)_2_D_3_) have been reported to be involved in the regulation of calcium and phosphorus utilization, skeletal mineralization, and osteogenic activity during embryonic development [57,58]. Furthermore, magnesium has been proposed to support these processes because of its dual role in growth plate chondrocyte proliferation and as a required cofactor for vitamin D hydroxylase enzymes [59,60]. These previously reported mechanisms may provide a biologically plausible explanation of the improvements in bone mineralization and mechanical strength observed in the present study. However, serum vitamin D metabolites, calcium-regulating hormones, gene expression, and bone turnover markers were not evaluated in the present study. Therefore, although our findings are consistent with these previously proposed mechanisms, the present study does not directly demonstrate the physiological and molecular pathways responsible for the observed responses. Future studies incorporating measurements of serum vitamin D metabolites, calcium-regulating hormones, gene expression, and bone turnover markers are warranted to verify the physiological and molecular mechanisms underlying the beneficial effects of in ovo vitamin D_3_ metabolite supplementation.

Regarding locomotor health, BA had no effect on GS, TD, or FHN, indicating that these traits are more strongly influenced by incubation and early post-hatch conditions than by parental age. Previous studies support limited BA effects on TD incidence [61] and emphasize the role of the incubation environment in locomotor outcomes [62]. FHN, on the other hand, is more closely associated with rapid growth and mechanical overload of skeletal tissues, often involving vascular disruption and bacterial involvement [63,64]. In the present study, vitamin D_3_ metabolites improved GS and reduced TD incidence. These effects were accompanied by improvements in bone mineralization and structural indices, suggesting that functional benefits were not isolated from skeletal development but part of a coordinated response. However, no effect was observed on FHN. Improvements in mineralization did not translate into lower FHN incidence, which is not unexpected given that FHN is influenced by vascular, mechanical, and bacterial factors in addition to skeletal integrity [63,64]. Although BA influenced selected skeletal traits, most of these differences were not evident at processing age. In contrast, IO administration of D_3_ metabolites consistently improved bone mineralization, mechanical properties, and locomotor-related traits across BA groups. The absence of significant BA × IO interactions for most skeletal traits suggests that the response to vitamin D_3_ metabolites was largely independent of maternal age. In practical terms, the benefits of vitamin D_3_ metabolite administration were expressed in offspring from both YB and OB flocks rather than being confined to a specific maternal age group. These findings suggest that optimizing embryonic vitamin D status may have a greater impact on skeletal quality than BA alone. Further studies conducted under commercial production conditions are warranted to determine whether the skeletal benefits observed under experimental conditions persist in environments characterized by greater biological and management-related stressors.

Previous studies have demonstrated beneficial effects of in ovo vitamin D supplementation on hatchability, post-hatch performance, and selected aspects of skeletal development [16,17,65]. The present study extends previous research by evaluating whether breeder age influences the skeletal response to in ovo vitamin D_3_ metabolite supplementation. By comparing broilers derived from young and old breeder flocks, the experimental design enabled assessment of the consistency of the skeletal response to vitamin D_3_ metabolites under different maternal conditions. Although breeder age affected several developmental traits, the improvements in bone mineralization and mechanical properties induced by vitamin D_3_ metabolites were consistently observed across breeder age groups. These findings extend current knowledge by showing that the beneficial skeletal effects of in ovo vitamin D_3_ metabolite supplementation were maintained across breeder age groups, thereby providing additional insight into embryonic nutritional programming.

## 5. Conclusions

In conclusion, BA had limited effects on tibia and femur traits and growth performance, whereas IO administration of D_3_ metabolites, particularly 25(OH)D_3_, consistently improved growth performance and leg health in broilers. These responses were associated with enhanced bone mineralization, as evidenced by increased tibial Ca, P, and Mg concentrations; higher bone ash content; a higher SI; and greater bone breaking strength. Accordingly, birds exhibited shorter and narrower but heavier long bones, indicating altered bone geometry rather than inherently improved skeletal development. In addition, IO administration of D_3_ metabolites reduced the incidence of tibial dyschondroplasia and gait abnormalities, whereas femoral head necrosis was unaffected. Overall, in ovo administration of vitamin D_3_ metabolites consistently enhanced bone mineralization and mechanical properties regardless of breeder age. These findings support the potential of in ovo vitamin D_3_ metabolite supplementation as a nutritional strategy to improve skeletal development under different maternal conditions.

## Figures and Tables

**Table 1:** Effect of breeder age and in ovo administration of vitamin D_3_ metabolites on egg weight, fertility and hatching performance.

Treatments		Egg Set (*n*)	Egg Weight (g)	Fertility (%)	Hatching Performance (%)		
					Hatchability of Fertile Eggs	Late Embryonic Mortality ^3^	Total Embryonic Mortality
Breeder age ^1^	YB	320	59.6 ^b^	88.44	85.81	1.89	13.19
	OB	320	67.3 ^a^	90.67	87.12	2.27	13.88
	SEM ^4^	-	0.178	0.125	0.136	0.141	0.161
In ovo ^2^	25(OH)D_3_	160	63.6	90.00	86.52	2.18	13.48
	1.25(OH)_2_D_3_	160	63.3	88.33	86.34	2.08	13.66
	Diluent	160	63.7	89.31	85.92	2.29	13.08
	Non-injected	160	63.2	90.60	87.08	1.81	13.92
	SEM ^4^	-	0.190	0.134	0.136	0.177	0.156
			*p*				
Breeder age (BA)			0.001	0.541	0.423	0.117	0.612
In ovo (IO)			0.749	0.623	0.275	0.092	0.108
BA x IO			0.361	0.274	0.157	0.121	0.165

a,b = Means within a column with different superscripts differ significantly (*p* < 0.05). **^1^** YB, young breeder (29 weeks old); OB, old breeder (52 weeks old). **^2^** In ovo: 25(OH)D_3_**:** Calsifediol dissolved in saline was injected into the amniotic fluid on E18 at a dose of 0.60 µg per egg in a volume of 100 μL containing 0.12% DMSO; 1,25(OH)_2_D_3_: Calcitriol dissolved in saline was injected into the amniotic fluid on E18 at a dose of 0.60 µg per egg in a volume of 100 μL containing 0.12% DMSO. Diluent: A total of 100 µL of saline containing 0.12% was injected into the amniotic fluid on E18 as the diluent. Non-injected: Eggs were not injected. **^3^** Late embryonic mortality: Embryonic mortality was observed between the 18th and 21st days of incubation. **^4^** SEM: Standard error of mean.

**Table 2:** Effects of breeder age and in ovo administration of D_3_ metabolites on body weight (BW, g), feed consumption (FC, g), and feed conversion ratio (FCR, g/g) of broiler chickens.

Treatments		BW (g)			FC (g)		FCR (g/g)	
		Hatch	21 d	42 d	0–21 d	0–42 d	0–21 d	0–42 d
Breeder age ^1^	YB	43.7 ^b^	762 ^b^	2773 ^b^	972 ^b^	4525 ^b^	1.276	1.632
	OB	47.8 ^a^	853 ^a^	2810 ^a^	1081 ^a^	4595 ^a^	1.267	1.635
	SEM ^3^	0.14	0.15	2.60	1.10	1.60	0.006	0.004
In ovo ^2^	25(OH)D_3_	46.1	820 ^a^	2820 ^a^	1038	4603 ^a^	1.266	1.632
	1,25(OH)_2_D_3_	45.8	809 ^ab^	2812 ^ab^	1028	4592 ^a^	1.271	1.633
	Diluent	45.6	802 ^b^	2760 ^b^	1023	4524 ^b^	1.276	1.639
	Non-injected	45.5	799 ^b^	2774 ^b^	1017	4521 ^b^	1.273	1.630
	SEM ^3^	0.14	0.25	0.78	1.20	1.60	0.006	0.004
		*p*						
Breeder age (BA)		0.001	<0.001	0.011	0.002	0.024	0.650	0.581
In ovo (IO)		0.096	<0.001	0.043	0.951	0.041	0.983	0.474
BA x IO		0.108	0.901	0.134	0.674	0.364	0.302	0.509

a,b = Means within a column with different superscripts differ significantly (*p* < 0.05). ^1^ YB, young breeder (29 weeks old); OB, old breeder (52 weeks old). ^2^ In ovo: 25(OH)D_3_: Calsifediol dissolved in saline was injected into the amniotic fluid on E18 at a dose of 0.60 µg per egg in a volume of 100 μL containing 0.12% DMSO; 1,25(OH)_2_D_3_: Calcitriol dissolved in saline was injected into the amniotic fluid on E18 at a dose of 0.60 µg per egg in a volume of 100 μL containing 0.12% DMSO. Diluent: A total of 100 µL of saline containing 0.12% was injected into the amniotic fluid on E18 as the diluent. Non-injected: Eggs were not injected. ^3^ SEM: Standard error of mean.

**Table 3:** Effects of breeder age and in ovo administration of vitamin D_3_ metabolites on morphometric traits and breaking strength of tibias and femurs in broilers.

Treatments		Tibia/Femur							
		Weight (g)	Length (mm)	Diameter (mm)	Breaking Strength (N)	Weight (g)	Length (mm)	Diameter (mm)	Breaking Strength (N)
Breeder age ^1^	YB	26.78	106.60	11.19	315	19.29	72.72 ^b^	11.34	352
	OB	26.87	107.05	11.29	307	19.37	75.58 ^a^	11.43	340
	SEM ^3^	0.045	0.562	0.138	4.491	0.036	0.364	0.145	4.762
In ovo ^2^	25(OH)D_3_	27.78 ^a^	105.94 ^b^	11.24	329 ^a^	20.05 ^a^	73.08 ^b^	11.61	360 ^a^
	1,25(OH)_2_D_3_	27.11 ^b^	105.23 ^b^	11.33	313 ^ab^	19.87 ^b^	73.54 ^b^	11.65	349 ^ab^
	Diluent	26.24 ^c^	107.26 ^a^	11.41	302 ^b^	18.97 ^c^	75.08 ^a^	11.25	338 ^b^
	Non-injected	26.17 ^c^	108.87 ^a^	10.98	300 ^b^	18.43 ^c^	74.90 ^a^	11.03	337 ^b^
	SEM ^3^	0.063	0.794	0.192	6.350	0.051	0.519	0.204	6.734
					*p*				
Breeder age (BA)		0.165	0.573	0.604	0.112	0.126	0.001	0.673	0.232
In ovo (IO)		<0.001	0.007	0.379	0.013	<0.001	0.001	0.073	0.041
BA x IO		0.060	0.157	0.385	0.498	0.547	0.433	0.115	0.970

a–c = Means within a column with different superscripts differ significantly (*p* < 0.05). ^1^ YB, young breeder (29 weeks old); OB, old breeder (52 weeks old). ^2^ In ovo: 25(OH)D_3_: Calsifediol dissolved in saline was injected into the amniotic fluid on E18 at a dose of 0.60 µg per egg in a volume of 100 μL containing 0.12% DMSO; 1,25(OH)_2_D_3_: Calcitriol dissolved in saline was injected into the amniotic fluid on E18 at a dose of 0.60 µg per egg in a volume of 100 μL containing 0.12% DMSO. Diluent: A total of 100 µL of saline containing 0.12% was injected into the amniotic fluid on E18 as the diluent. Non-injected: Eggs were not injected. ^3^ SEM: Standard error of mean.

**Table 4:** Effects of breeder age and in ovo administration of vitamin D_3_ metabolites on tibia mineral concentrations and bone ash content in broilers.

Treatments		Tibia (%)				Femur (%)
		Ca	P	Mg	Ash	Ash
Breeder age ^1^	YB	32.42 ^a^	15.45 ^a^	0.856 ^a^	30.77	30.96
	OB	32.08 ^b^	15.26 ^b^	0.846 ^b^	30.43	30.60
	SEM ^2^	0.093	0.055	0.001	0.495	0.378
In ovo ^2^	25(OH)D_3_	32.78 ^a^	15.61 ^a^	0.866 ^a^	31.01 ^a^	31.25 ^a^
	1.25(OH)_2_D_3_	32.49 ^a^	15.51 ^a^	0.861 ^a^	30.91 ^a^	31.16 ^a^
	Diluent	31.96 ^b^	15.19 ^b^	0.837 ^b^	30.21 ^b^	30.41 ^b^
	Non-injected	31.77 ^b^	15.11 ^b^	0.840 ^b^	30.27 ^b^	30.30 ^b^
	SEM ^3^	0.131	0.002	0.002	0.699	0.535
		*p*				
Breeder age (BA)		0.011	0.018	0.001	0.111	0.773
In ovo (IO)		0.001	0.001	0.001	0.001	0.013
BA x IO		0.394	0.719	0.514	0.741	0.629

a,b = Means within a column with different superscripts differ significantly (*p* < 0.05). ^1^ YB, young breeder (29 weeks old); OB, old breeder (52 weeks old). ^2^ In ovo: 25(OH)D_3_: Calsifediol dissolved in saline was injected into the amniotic fluid on E18 at a dose of 0.60 µg per egg in a volume of 100 μL containing 0.12% DMSO; 1,25(OH)_2_D_3_: Calcitriol dissolved in saline was injected into the amniotic fluid on E18 at a dose of 0.60 µg per egg in a volume of 100 μL containing 0.12% DMSO. Diluent: A total of 100 µL of saline containing 0.12% was injected into the amniotic fluid on E18 as the diluent. Non-injected: Eggs were not injected. ^3^ SEM: Standard error of mean.

**Table 5:** Effects of breeder age and in ovo administration of vitamin D_3_ metabolites on gait score (GS), tibial dyschondroplasia (TD), and femoral head necrosis (FHN) in broilers.

Treatments		GS	TD	FHN
Breeder age ^1^	YB	0.203	1.292	0.408
	OB	0.319	1.125	0.258
	x ^2^	4.178	1.821	4.987
	*p*	0.124	0.610	0.173
In ovo ^2^	25(OH)D_3_	0.117	0.417	0.167
	1.25(OH)_2_D_3_	0.167	0.917	0.083
	Diluent	0.407	1.500	0.333
	Non-injected	0.353	2.000	0.749
	x ^2^	15.753	32.643	10.140
	*p*	0.015	<0.001	0.339

^1^ YB, young breeder (29 weeks old); OB, old breeder (52 weeks old). ^2^ In ovo: 25(OH)D_3_: Calsifediol dissolved in saline was injected into the amniotic fluid on E18 at a dose of 0.60 µg per egg in a volume of 100 μL containing 0.12% DMSO; 1,25(OH)_2_D_3_: Calcitriol dissolved in saline was injected into the amniotic fluid on E18 at a dose of 0.60 µg per egg in a volume of 100 μL containing 0.12% DMSO. Diluent: A total of 100 µL of saline containing 0.12% was injected into the amniotic fluid on E18 as the diluent. Non-injected: Eggs were not injected.

## Data Availability

Data used in the current study are available from the authors on request.

## Supplementary Materials

The following supporting information can be downloaded at https://www.mdpi.com/article/10.3390/ani16142229/s1: Figure S1: Distribution of gait scores (%) in broilers as influenced by breeder age and in ovo administration of vitamin D_3_ metabolites. Figure S2: Distribution of tibial discondraplasia (%) in broilers as influenced by breeder age and in ovo administration of vitamin D_3_ metabolites. Figure S3: Distribution of femoral head necrosis (%) in broilers as influenced by breeder age and in ovo administration of vitamin D_3_ metabolites.

## Abbreviations

ADU	Aydın Adnan Menderes University
BA	Breeder age
BW	Body weight
FC	Feed consumption
FCR	Feed conversion ratio
FHN	Femoral head necrosis
GS	Gait score
IO	In ovo
OB	Old breeder (52 weeks)
RH	Relative humidity
RI	Robusticity index
SI	Seedor index
TD	Tibial dyschondroplasia
wk	Week
YB	Young breeder (29 weeks)
