# Supplementary material for: Effects of Breeder Age and In Ovo Administration of Vitamin D3 Metabolites on Hatchability, Growth Performance, Bone Quality, and Leg Health in Broilers
Source: Animals (Basel). 2026 Jul 18;16(14):2229. doi: 10.3390/ani16142229 (PMC13405616; doi:10.3390/ani16142229)
Supplement: Supplementary file 1 [file animals-16-02229-s001.zip › animals-4409306-supplementary.pdf]

Supplementary Materials

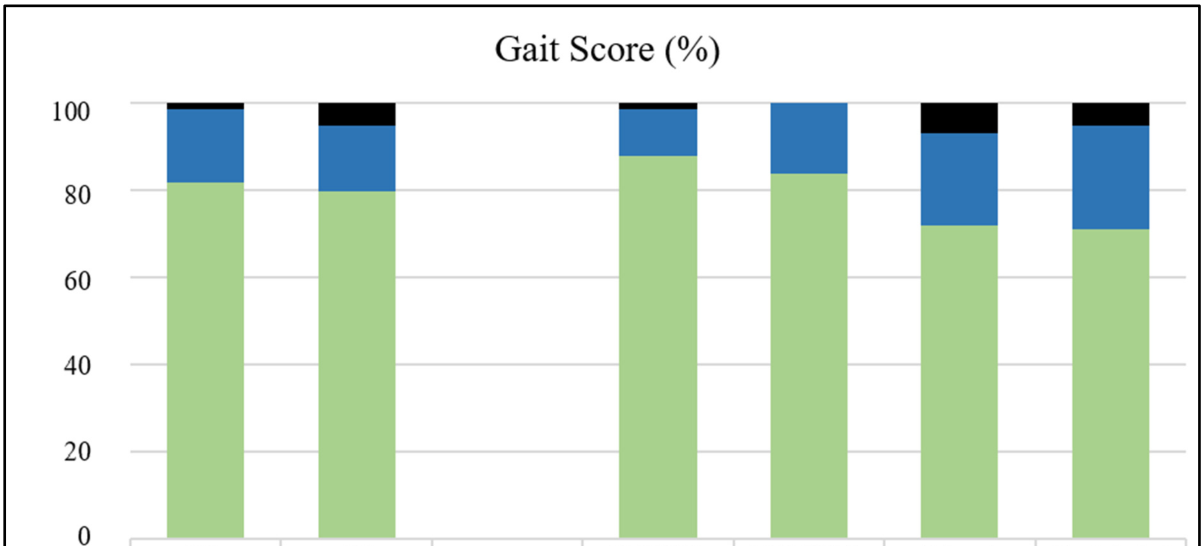

	YB	OB		25(OH)D₃	1,25(OH)₂D₃	Diluent	Noninjected
GS 2	1,4	5,0		1,2	0,0	6,9	5,2
GS 1	16,7	15,3		11,0	16,2	21,0	23,7
GS O	81,9	79,7		87,8	83,8	72,1	71,1

Figure S1: Distribution of gait scores (%) in broilers as influenced by breeder age and in ovo administration of vitamin D_3 metabolites.

GS: Gait score (0: no impairment of walking ability; 1: having obvious impairment but still ambulatory; 2: having severe impairment and not able to walk without great difficulty),

YB, young breeder (29 weeks old); OB, old breeder (52 weeks old)

25(OH)D_3: Calsifediol dissolved in saline was injected into the amniotic fluid on E18 at a dose of 0.60 µg per egg in a volume of 100 µL containing 0.12% DMSO, **1,25$(OH)_2D_3$:** Calcitriol, dissolved in saline was injected into the amniotic fluid on E18 at a dose of 0.60 µg per egg in a volume of 100 µL containing 0.12% DMSO, **Diluent:** A total of 100 µL of saline containing 0.12% was injected into the amniotic fluid on E18 as the diluent, **Non-injected:** Eggs were not-injected

Breeder age: $x^2 = 4.178$, P=0.124

In ovo: $x^2 = 15.753$, P=0.015

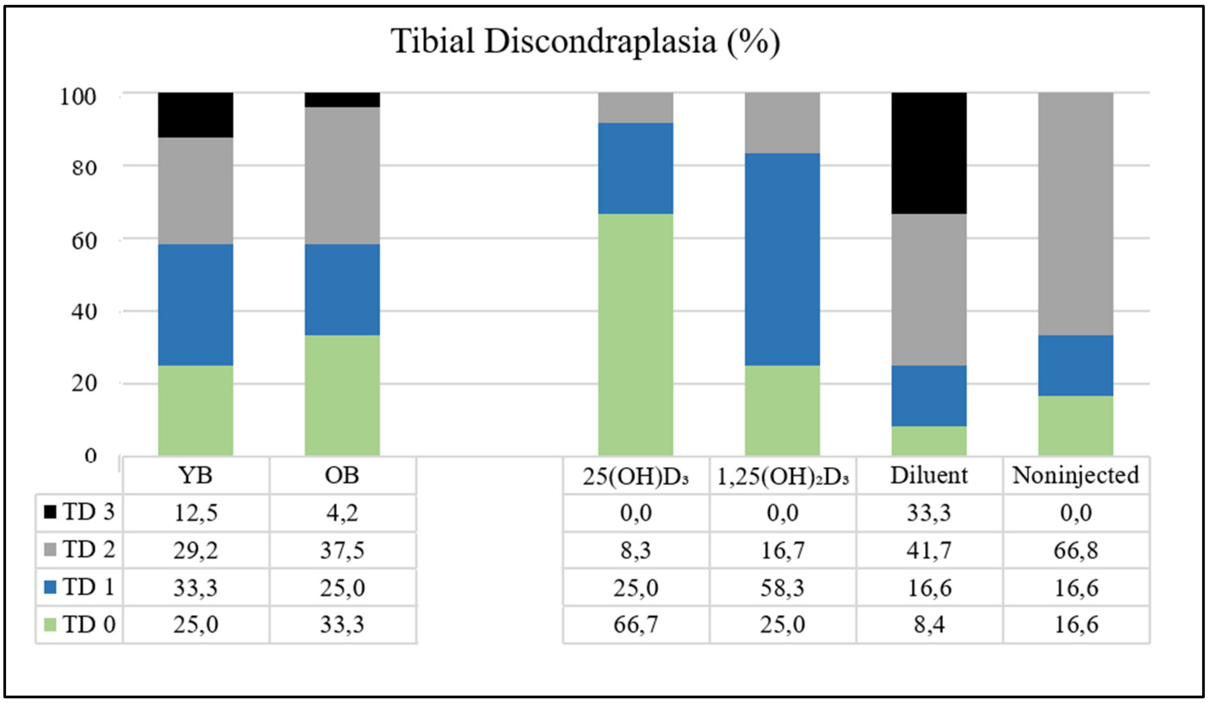

	YB	OB	25(OH)D₃	1,25(OH)₂D₃	Diluent	Noninjected
TD 3	12,5	4,2	0,0	0,0	33,3	0,0
TD 2	29,2	37,5	8,3	16,7	41,7	66,8
TD 1	33,3	25,0	25,0	58,3	16,6	16,6
TD 0	25,0	33,3	66,7	25,0	8,4	16,6

Figure S2: Distribution of tibial discondraplasia (%) in broilers as influenced by breeder age and in ovo administration of vitamin D_3 metabolites.

TD: Tibial Discondraplasia (0: no apparent TD, 1: growth plates 1 to 2 mm wide, 2: growth plates 2 to 3 mm wide, 3: growth plates greater than 3 mm wide),

YB, young breeder (29 weeks old); OB, old breeder (52 weeks old)

25(OH)D_3: Calsifediol dissolved in saline was injected into the amniotic fluid on E18 at a dose of 0.60 µg per egg in a volume of 100 µL containing 0.12% DMSO, **1,25$(OH)_2D_3$:** Calcitriol, dissolved in saline was injected into the amniotic fluid on E18 at a dose of 0.60 µg per egg in a volume of 100 µL containing 0.12% DMSO, **Diluent:** A total of 100 µL of saline containing 0.12% was injected into the amniotic fluid on E18 as the diluent, **Non-injected:** Eggs were not-injected

Breeder age: x^2 = 1.821, P= 0.610

In ovo: x^2 =32.643, P= <0.001

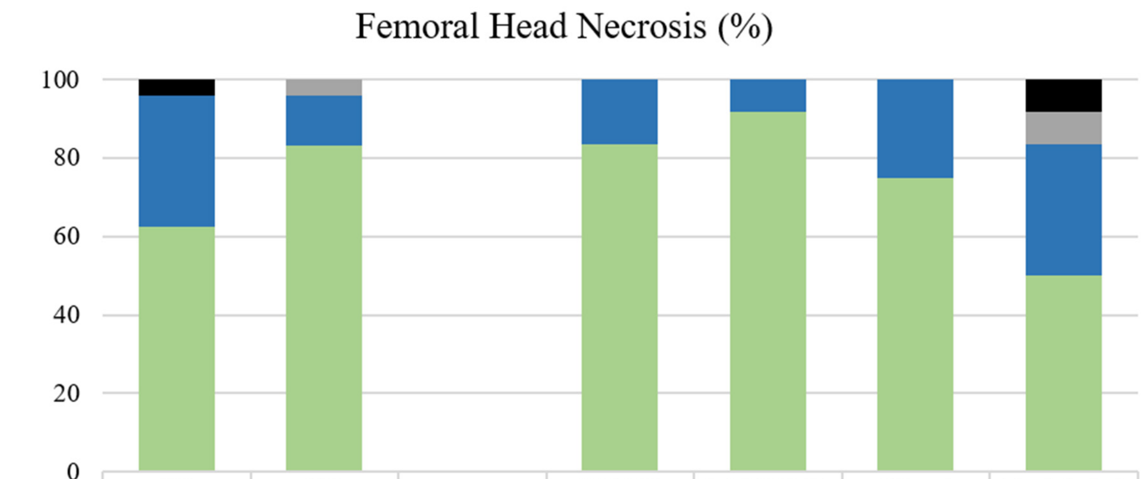

	YB	OB	25(OH)D₃	1,25(OH)₂D₃	Diluent	Noninjected
FHN 3	4,2	0,0	0,0	0,0	0,0	8,3
FHN 2	0,0	4,2	0,0	0,0	0,0	8,3
FHN 1	33,3	12,5	16,6	8,3	25,0	33,4
FHN 0	62,5	83,3	83,4	91,7	75,0	50,0

Figure S3: Distribution of femoral head necrosis (%) in broilers as influenced by breeder age and in ovo administration of vitamin D_3 metabolites.

FHN: Femoral Head Necrosis (0: no abnormalities (normal), 1: separation of the head from the acetabulum, 2: transitional degeneration, 3: severe necrosis),

YB, young breeder (29 weeks old); OB, old breeder (52 weeks old)

25(OH)D_3: Calsifediol dissolved in saline was injected into the amniotic fluid on E18 at a dose of 0.60 µg per egg in a volume of 100 µL containing 0.12% DMSO, **1,25(OH)$_2D_3$:** Calcitriol, dissolved in saline was injected into the amniotic fluid on E18 at a dose of 0.60 µg per egg in a volume of 100 µL containing 0.12% DMSO, **Diluent:** A total of 100 µL of saline containing 0.12% was injected into the amniotic fluid on E18 as the diluent, **Non-injected:** Eggs were not-injected

Breeder age: x^2= 4.987, P=0.173

In ovo: x^2= 10.140, P=0.339
